# Distance mathematics teaching in Flanders, Germany, and the Netherlands during COVID-19 lockdown

**DOI:** 10.1007/s10649-021-10094-5

**Published:** 2021-10-14

**Authors:** Paul Drijvers, Daniel Thurm, Ellen Vandervieren, Marcel Klinger, Filip Moons, Heleen van der Ree, Amy Mol, Bärbel Barzel, Michiel Doorman

**Affiliations:** 1 Utrecht University, Utrecht, the Netherlands; 2 Universität Duisburg-Essen, Essen, Germany; 3 University of Antwerp, Antwerp, Belgium

**Keywords:** Corona pandemic, COVID-19, Didactical approaches, Distance assessment, Distance education, Mathematics education, Teacher beliefs, Teaching at distance, Teaching practices

## Abstract

The COVID-19 pandemic has confronted mathematics teachers with the challenge of developing alternative teaching practices—in many cases at a distance through digital technology—because schools were closed. To investigate what distance practices in secondary mathematics education have emerged and how teachers experienced them, we set out online questionnaires in Flanders—the Dutch-speaking part of Belgium—, Germany, and the Netherlands. The questionnaire focused on teaching practices, teacher beliefs, didactics, and assessment. Data consisted of completed questionnaires by 1719 mathematics teachers. Results show that the use of video conferencing tools increased massively, while the use of mathematics-specific tools that teachers used before the lockdown reduced substantially. Further findings are that teachers' confidence in using digital technologies increased remarkably during the lockdown and that their experiences and beliefs only marginally impacted their distance learning practices. Also, we observed some differences between the three countries that might be explained by differences in educational policies and in technological facilities and support. For future research, it would be relevant to investigate long-term changes in teachers’ practices, as well as students’ views and experiences related to the teacher’s practices.

## Introduction

Since the spring of 2020, the COVID-19 viral pandemic has drastically disrupted the international community worldwide. After an initial period of confusion, rapid transitions have taken place in society and economy. The pandemic has also clearly impacted education, which has been confronted with critical measures such as home confinements in many countries. Schools in many countries have been closed, leaving teachers with the challenge of developing alternative teaching practices, in many cases at a distance through digital technology. At the time of writing this paper, this process of developing new teaching practices is ongoing.

Mathematics education, of course, has also been affected by this crisis. Whereas mathematics teachers in the past may have been reluctant to change their teaching practices, for example concerning the integration of digital technology, school lockdowns have thrown them en masse into a steep learning curve on practices for distance education. Mathematics education having its specific characteristics and demands, such as an emphasis on interaction and reinvention, and the need for specific representations like formulas and graphs, it is highly relevant to monitor the first experiences with the emerging practices in this situation of crisis.

Some recent literature sheds some first light on these emerging practices. Aldon et al. (2021) showed that mathematics teachers in France, Israel, Italy, and Germany were facing challenges in teaching at distance with respect to supporting students’ learning, developing assessment, supporting students who face difficulties, and exploiting potentialities for fostering typical mathematical processes. Hodgen and colleagues (Hodgen et al. (2020) interviewed heads of departments in the UK and found that student–teacher interaction in mathematics teaching at distance was a concern. Clark-Wilson et al. (2020) claimed that mathematics teachers in many countries were unprepared for online teaching. The Nesta (2020) report suggested that COVID led to continuing and even widening gaps between students in terms of their engagement and access to technology. Overall, there is a concern of students’ learning loss (Engzell et al., 2020), and recent policy documents highlight the need for new teaching structures, practices, and advocacy in mathematics education (NCSM & NCTM, 2020).

Still, much remains unknown about the mathematics teaching practices that have emerged in times of school closure. The topic at stake in the study presented here, therefore, is what distance teaching practices in mathematics have emerged in the spring 2020 period of school lockdown and how teachers experienced these drastic changes; a topic that is highly relevant to educators, researchers, and policy makers, also in the light of possible future pandemics. In particular, we wanted to investigate the emerging practices of mathematics education at distance, the relationship between these practices and teachers’ beliefs on mathematics teaching and learning, the impact of distance education on didactical approaches to mathematics, and emerging assessment practices.

We investigated these topics through a theory-based online questionnaire among mathematics teachers in three neighboring, Western-European countries or regions in which schools closed in spring 2020: Flanders—the Dutch-speaking part of Belgium—Germany, and the Netherlands. In what follows, we describe the theoretical background, the setup and the administration of the questionnaire, and its results.

## Theoretical background

In the conception phase of this study, we identified four perspectives that, in interplay, may be helpful in describing a teacher’s preparation and delivery of teaching practices at distance: (1) instrumental orchestration as a means to capture the way teaching practices are set up, (2) the teacher’s beliefs on mathematics education and the role of digital technology in it, (3) the teacher’s didactical ideas on how and what to teach, and (4) the opportunities for formative and summative assessment. Therefore, the theoretical background of the study includes these four perspectives to describe and understand the teaching practices. Below, we address each of these perspectives in more detail.

### Instrumental orchestration

Instrumental orchestration, the first theoretical lens, acknowledges that learning and teaching mathematics with and through technology requires a rethinking and a re-arrangement of traditional teaching formats. As such, it can be used to identify and describe teaching practice. Stein and colleagues describe orchestrations that focus on productive mathematical discussions (Stein et al., 2008), which might be challenging to set up in education at distance. Bozkurt and Ruthven (2018) particularly address activity structures in mathematics teaching while using digital technology. Recently, NCSM and NCTM (2020) provide guidelines for teaching practices for the case of distance education in times of COVID-19.

An instrumental orchestration (IO) is defined as the teacher’s intentional and systematic organization and use of the various artifacts available in a—in this case computerized—learning environment in a mathematical task situation (Trouche, 2004). We distinguish three elements within an instrumental orchestration: a didactical configuration, an exploitation mode, and a didactical performance (Drijvers et al., 2010). A didactical configuration is an arrangement of artifacts in the environment, a configuration of the teaching setting, and the artifacts involved in it (Drijvers et al., 2020). An exploitation mode is the way the teacher decides to exploit a didactical configuration for the benefit of the didactical intentions. A didactical performance involves the ad hoc decisions taken while teaching on how to perform in the chosen didactical configuration and exploitation mode.

In this study, we used the IO model in the design of questionnaire items on the emerging teaching practices, from a didactical configuration perspective (“how to set up the teaching”), and an exploitation mode perspective (“how to use the setting”). Seen from this theoretical lens, we conjecture that teachers in the school lockdown period would set up new configurations and exploitation modes.

### Teacher beliefs

Teachers’ instrumental orchestrations of digital technology are closely associated with their beliefs (Thomas & Palmer, 2014), which is the second theoretical lens of the study. Beliefs can be described as “psychologically held understandings, premises, or propositions about the world that are thought to be true” (Philipp, 2007, p. 259). Teachers’ beliefs impact on their practices, as they filter and frame, provide an orienting and guiding function, and connect knowledge and action (Fives & Buehl, 2012). Therefore, teachers who believe in the value of digital technology for teaching and learning process are more likely to incorporate technology into their practice (Ertmer & Ottenbreit-Leftwich, 2010). In addition, teachers need self-efficacy beliefs for teaching with digital technology (Thomas & Palmer, 2014; Thurm & Barzel, 2019), described as “beliefs in one’s capabilities to organize and execute the courses of action required to produce given attainments” (Bandura, 1997, p. 3). Teachers’ high self-efficacy beliefs are associated with incorporating digital technology and holding more positive beliefs about its value for teaching mathematics (Thomas & Palmer, 2014; Thurm & Barzel, 2019).

Teacher beliefs can vary in strength and are part of a dynamic belief system (Philipp, 2007). Strongly held beliefs usually have been shaped over a long period of time and are grounded in substantial personal experience. Changing such beliefs is considered difficult. New personal experiences are the strongest impetus for change in teachers’ beliefs. For example, teachers’ self-efficacy for teaching mathematics with technology may change through the experience of being able to successfully implement digital technology in their classroom (Ertmer & Ottenbreit-Leftwich, 2010).

Most studies so far have addressed teacher beliefs in classical teaching settings (e.g., Pierce & Ball, 2009; Thurm, 2018). In the study presented here, we focused on teacher beliefs about mathematics distance education. Because many teachers were pushed into distance learning, and their beliefs about distance learning were not yet deeply rooted, we expected changes in beliefs due to their distance teaching experiences. We conjectured that the type of change (more positive or more negative) might depend on factors such as school support, technical infrastructure, previous experience with digital technology, and frequency of online distance teaching. These ideas are reflected in the questionnaire items on teacher beliefs.

### Didactical approaches to distance mathematics education

The third theoretical lens guiding this study concerns didactical approaches to (distance) mathematics education. Many teachers, educators, and researchers share the view that the goals of mathematics education go beyond training basic procedural skills and include higher-order competences such as mathematical thinking, conceptual understanding, and processes such as problem solving, modeling, and reasoning. To address these competences, different didactical approaches have been suggested. Freudenthal (1991) suggested guided reinvention as an underlying principle of a Realistic Mathematics Education approach. More recently, inquiry-based learning (IBL) was developed as a means to focus on these higher-order learning goals. IBL refers to a teaching approach in which students work at their level of competence “to do inquiry” in unstructured problem situations (Swan et al., 2013). In contrast to direct instruction, the IBL-approach allows students to take responsibility for their learning as they work individually or in groups while developing new mathematical insights (Barzel, 2005). Authentic problem situations may be used to invite students’ engagement in modeling processes.

Many of these didactical approaches are student-centered and ask for interactive teaching formats, in which the role of the teacher is not straightforward, since results of students’ inquiry can be quite diverse, and unexpected classroom situations may arise (Dobber et al., 2017). The teacher should ensure, using a variety of solutions to open and model-eliciting tasks, to foster a shared understanding of general mathematical concepts and procedures, which is not an easy task (e.g., Doorman, 2019; Gravemeijer, 2004).

Distance teaching may limit student and teacher interaction, and, for example, the opportunities to observe students’ work to notice potentialities (Stillman, 2019). In online settings, the teacher’s guidance is limited, or at least teachers are hardly experienced in exploiting the potential of open-ended tasks and guidance-related challenges in online environments. Therefore, we used the above theories on new didactical approaches in the study presented here as a lens to find out whether teachers in the situation of distance education have changed their didactical approach and focused on procedural basic skills and practices rather than on higher-order learning goals.

### Assessment

The fourth theoretical lens, assessment, has always been a topic of interest to both teachers and students. Traditionally, summative assessment, which includes high-stakes tests in which student learning and skill acquisition are evaluated, is considered highly relevant. More recently, formative assessment, which concerns the response “on the fly” to student processes to enhance further learning, receives much attention as well (e.g., see Wiliam, 2011). Both summative and formative assessments are at the heart of teachers’ practices and are subject to changes if face-to-face education changes into distance education.

Stacey and Wiliam (2013) distinguished two types of technology-rich summative assessment, which Drijvers et al. (2016) labeled “assessment with technology” and “assessment through technology.” In assessment with technology, the test may have a traditional paper-and-pen format, but additional use of technology, such as a calculator, is included. In such cases, the technology serves as an add-on to the traditional assessment setting. In assessment through technology, however, the test is delivered and administered through technological means. Technology is the vehicle and provides the environment in which the assessment takes place. Online tests are a typical example of assessment through technology. Whereas mathematics teachers may be experienced in summative assessment with technology—using a calculator during an examination is quite common—we conjecture that they may be less familiar with assessment through technology, which might be a natural option in the distance education scenario.

For technology-enhanced formative assessment, the following key strategies have been identified (Ruchniewicz & Barzel, 2019; Wiliam & Thompson, 2008): clarifying learning intentions, engineering classroom discussion & learning tasks that elicit student understanding, providing feedback to move learners forward, activating students as resources for one another, and activating students as owners of their learning. We conjectured that teachers might be hindered in using these strategies to provide formative feedback to their students by the limited means of communication available in the technological environments.

### Research questions

Responding to the challenges that mathematics teachers in our countries have been facing recently, the study’s central research question is: Which distance teaching practices in secondary mathematics education have emerged in Flanders, Germany, and the Netherlands in the spring 2020 period of school lockdown and how have teachers experienced them?

In a one-to-one correspondence to the four theoretical perspectives addressed above, we phrased the following sub-questions, each with some hypotheses.

1. Which distance teaching practices do mathematics teachers shape, and how do they experience them?
  The instrumental orchestration model highlights the intertwinements of opportunities and constraints of the technological infrastructure and support on the one hand, and the didactical configurations and the exploitation modes that teachers enact. Therefore, we would expect a correlation between infrastructure level and, for instance, the use of synchronous or asynchronous orchestrations.
2. How do emerging teaching practices relate to mathematics teachers’ beliefs?
  We conjecture that emerging teaching practices relate to the teachers’ beliefs, and to their confidence in and experience with using digital technology in their teaching. In particular, a positive attitude towards digital technology in mathematics teaching is expected to become even more positive.
3. Which didactical approaches arise during the distance mathematics education?
  In line with past experiences (e.g., see Rudd, 2007, on the integration of interactive whiteboards), we expect teachers to focus on “safe and easy” approaches to their teaching, that is, to rehearsing and practicing mathematical procedures, rather than to focus on higher-order learning goals such as mathematical understanding, and on new mathematical topics.
4. What assessment formats are used and what are their opportunities and limitations for teachers?
  In terms of the summative-formative dimension and the with-through distinction, we expect teachers to feel somewhat hindered in providing formative feedback to their students through digital means because of the limited means of communication, and to struggle with formats for summative assessment through technology.

Of course, the four perspectives described above act in interplay. We conjectured that school infrastructure and support might relate to the use of (a)synchronous orchestrations, as synchronous formats put more demands on technological infrastructure. Also, we expected a relationship between teachers’ beliefs and confidence on the one hand, and their use of synchronous teaching formats and their didactical approaches on the other: would it not seem likely that a high level of confidence helps in setting up more challenging orchestrations involving new mathematical topics, conceptual understanding, and student–teacher interaction?

## Methods

To address the above research questions, we set up an online questionnaire in Flanders (FL), Germany (GE), and the Netherlands (NL) among mathematics teachers in secondary education, as we considered this to be the most feasible and efficient way to attract a large number of respondents. Through describing and comparing the responses in the three countries and regions—from now on just called “countries”—we wanted to answer the research questions. We also administered a similar questionnaire among these teachers’ students; in this paper, we only report on the teachers’ responses.

### Research context in the three participating countries

**Fig. 1:**
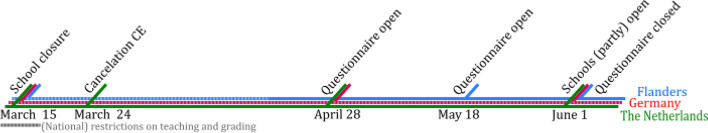
Timeline for school lockdown and questionnaires in 2020 in the three countries

We chose to include these three countries in our study because of their interesting similarities and differences. As for the similarities, Flanders, Germany, and the Netherlands are adjacent countries in Western-Europe and share an educational system of primary and secondary school, where the latter includes students of 12–18, or in Germany 10–18-year-old. The three countries also have had quite similar COVID-19 pandemic restriction policies. In particular, all three took the political decision to close secondary schools on March 15, 2020, until early June (see Fig. 1).

Despite these common characteristics of the three geographically close countries, there are important institutional differences. Whereas Flanders and the Netherlands each have a nationwide educational system, Germany’s federal structure includes sixteen states (the so-called Bundesländer), each with its own educational system. In at least some of them, the local ministries of education suggested the teachers to focus on rehearsing and practicing during the closing of schools. Furthermore, in some German states, students’ performance during school closure could not be used for grading purposes. In Flanders, teachers were obliged to rehearse already seen content until the Easter holidays (19th of April). After the Easter holidays, schools were encouraged to start providing new materials to students. As Flemish education is organized in a decentralized way, schools could decide freely whether they would re-open at the end of May and if and how they would organize final exams. In the Netherlands, the ministry of education decided that national central final examinations (CE) would be canceled, and that students would receive their secondary school diploma based on previously administered school-based assessments.

### Instrument development: the questionnaire

The study’s main instrument was a questionnaire for mathematics teachers. As a consequence, the study is based on teachers’ self-reports. We designed the questionnaire in English and translated it into German and Dutch. Although Dutch is spoken in the Netherlands as well as in Flanders, the questionnaires were localized according to differences in available technology, in commonly used vocabulary, et cetera. Despite these localizations, we carefully maintained the common meaning of questionnaire items. Appendix 1 shows the English version of the questionnaire, after the deletion of items that, for instance, concern the consent to participate.

For each of the four research perspectives reflected in the sub-questions—practices, beliefs, didactics, and assessment—we designed questionnaire items. Appendix 2 (Table 9) shows an overview of the questionnaire items and their correspondence to the research sub-questions. As we aimed to get a global picture of all these aspects and in the meantime would not want to ask too much of the respondents’ time to fill in the questionnaire, many of the topics are addressed in only one item. As item types, we used multiple-choice, multiple-response, Likert-type items, slider bars, and open response items. Most items referring to teachers’ confidence had a 0–100 scale format, as recommended by Bandura (2006).

As a questionnaire for this pandemic situation did not exist, we designed its items, based on the literature referred to in Sect. 2, which was expected to provide content validity. In addition, an earlier version of the questionnaire was piloted among a limited number of teachers in the three participating countries, and the feedback led to clarifications in the final version.

### Questionnaire administration

The questionnaire was implemented in Qualtrics,^1^ a system that allows for digitally signing for consent. The study setup was approved by Utrecht University’s Science-Geo Ethics Review Board (for German and Dutch data) and the University of Antwerp’s Ethics Committee for the Social Sciences and Humanities (for Flemish data). Overall guidelines for privacy and data management were respected.

The online questionnaire was released on April 28, 2020, in Germany and the Netherlands, and on May 18, in Flanders, and was closed in all countries on June 1 (see Fig. 1). The invitation to take part was communicated to mathematics teachers through professional online newsletters, direct mails to members of associations of mathematics teachers, dedicated social media groups, teacher association websites, messages to school principals, et cetera. As a minor incentive, teachers were promised to receive a report on the findings through email after their participation and our data analysis.

### Participants

In total, 2616 teachers responded to the invitation to fill in the questionnaire. However, only 1719 of them (about two-third) finished the questionnaire, in the sense that they opened the final item—which does not imply that all items are answered. As many of the items in the uncompleted questionnaires were not answered, we decided to only include and analyze the 1719 finished ones. Because of the voluntary participation of the teachers, results need to be interpreted with caution as they might not be representative for all the mathematics teachers in each country. Based on responses to items on teachers’ beliefs, it seems that teachers who are enthusiastic about their new teaching practices might be over-represented. Still, the topic of how to teach in the lockdown situation was such an important issue to teachers that we expect that teachers who felt badly about it also shared their experiences, which was reflected in some of the responses.

Of the 1719 included responses, 384 (22%) came from Flanders, 1131 (66%) from Germany, and 204 (12%) from the Netherlands. Relative to the countries’ populations (6.6 M for FL, 17.3 M for NL, and 83.0 M for GE), Flanders has the highest response. Table 1 provides information on the gender distribution of the included respondents. The majority of the teachers is female, which seems to reflect the gender ratio of secondary mathematics teachers nationwide in Flanders (68%), of all secondary teachers in Germany (50%), and to a lesser extent for the Netherlands, where 42% of the mathematics teachers nationwide is female. Overall, the Dutch respondents (mean age of 48.6 year) are slightly older than their Flemish (44.0) and German (42.3) colleagues.

**Table 1:** Respondents’ gender distribution in the participating countries

Gender	Flanders	Germany	The Netherlands	Total
Female	287 (75%)	637 (56%)	113 (55%)	1037 (60%)
Male	96 (25%)	485 (43%)	90 (44%)	671 (39%)
Other	1 (0%)	2 (0%)	0 (0%)	3 (0%)
Missing	0 (0%)	7 (1%)	1 (1%)	8 (1%)
Total	384 (100%)	1131 (100%)	204 (100%)	1719 (100%)

### Data analysis

After the online questionnaire was closed, data from the three countries were merged, anonymized, and exported to SPSS. As a first step in the data analysis, we addressed each of the four perspectives and the corresponding research sub-questions (see the Table 9 in Appendix 2).

As most topics were addressed in just one item, we carried out an item-by-item analysis. Conceptually, a single item “lends itself to absolute clarity and transparency about what is being measured” (Postmes et al., 2013, p. 598). Research has shown that single-item measures can have reasonably high reliabilities, depending on the nature of the construct operationalized (Postmes et al., 2013; Wanous & Hudy, 2001). The item-by-item analyses initially concentrated on describing and exploring the data. The intermediate results were shared and discussed in regular video conferences with the whole team. Some items were recoded. To allow for comparison with non-Likert items, we dichotomized responses to some of the Likert-type items. For example, responses to item T3 on tools used before lockdown were recoded into users (responses “twice per month,” “once per week,” “daily”) and non-users (responses “not” and “once”), to facilitate comparison with the responses to item T4 on practices during lockdown. Also, a variable T3_Total was created as an indicator of overall mathematical tool use. To enable comparison between T20 and T21 on changes in confidence in distant formative assessment practices, we rescaled T21’s scores into percentages.

Next, correlations were calculated for items within these themes, to check out the initial hypotheses phrased in Sect. 2.5. In line with recommendations from literature, and because the Likert data showed many ties, we used Kendall’s Tau-*b* to measure ordinal correlations (Croux & Dehon, 2010). Even if correlation values should be interpreted in context, as an overall starting point, we considered Tau-*b* values of 0.2 or higher as suggesting a moderate but relevant relationship (for a comparison, see Akoglu, 2018). Finally, we carried out some cross-over analyses involving the four different perspectives to identify correspondences across them.

## Results

In this section, we summarize the main results according to the four research sub-questions on teaching practices, teacher beliefs, didactics, and assessment. In Sect. 4.5, we present the results across these perspectives to answer our initial hypotheses.

### Teaching practices at a distance

To describe the teaching practices in times of school lockdown, we distinguish how the teachers set up their teaching (cf. the didactical configuration in the IO model) and the ways in which they used this setup (cf. the IO exploitation mode).

To get more insight into how teachers set up their distance teaching, we asked them how they delivered their math teaching before and since the schools were closed (questionnaire items T3 and T4). Of the German teachers, 3% indicated they did not teach anymore since the school lockdown. In Flanders and the Netherlands, all respondents were still teaching. Table 2 shows which tools were used for delivering the teaching in each country before and since school closure. For each item, the percentage of teachers who use the tool is reported.

**Table 2:** Delivery tools before and since school lockdown in the participating countries (items T3 and T4, *N* = 1719)

	Flanders (*n* = 384)		Germany (*n* = 1131)		The Netherlands (*n* = 204)	
	Before	Since	Before	Since	Before	Since
Video conferencing software	4%	87%	2%	56%	3%	97%
Social media	12%	6%	5%	9%	12%	7%
Learning management system	57%	68%	31%	56%	94%	75%
Online video clips	27%	41%	52%	61%	60%	59%
Online exercise platforms	37%	19%	14%	19%	35%	14%
Online learning environments	55%	25%	49%	26%	50%	16%
Homemade video clips	11%	67%	8%	33%	14%	28%
Audience response systems	27%	13%	19%	10%	35%	27%

In each of the three countries, math teachers indicate a strong increase in the use of video conferencing software, but to a lesser extent in Germany than in Flanders and the Netherlands. In Flanders, and to a lesser extent in Germany and the Netherlands, there is also a big increase in the use of homemade video clips. After the schools were closed, online learning environments (e.g., Desmos, DWO, GeoGebra Books, GeoGebra Tube, Math4All) and audience response systems (e.g., Socrative, Mentimeter, Kahoot!) have been used less in the three countries.

When it comes to the use of online exercise platforms, online video clips, social media, and learning management systems, some discrepancies between the countries can be noted.

- The use of online exercise platforms (e.g., AlgebraKit, Bettermarks, DWO, Sowieso, software from the textbook publishing company) decreased remarkably in the Netherlands and Flanders while it slightly increased in Germany.
- The use of already available online video clips increased the most in Flanders, where online video clips had been used only rarely before the pandemic. An increase can also be observed for Germany, while no important change was observed for the Netherlands.
- Social media use for mathematical learning decreased in Flanders and the Netherlands whereas it slightly increased in Germany. The use of learning management systems increased in Flanders and Germany while it dropped in the Netherlands, probably because learning management systems had been used by a wide group of teachers in the Netherlands already before the crisis (94%).

**Fig. 2:**
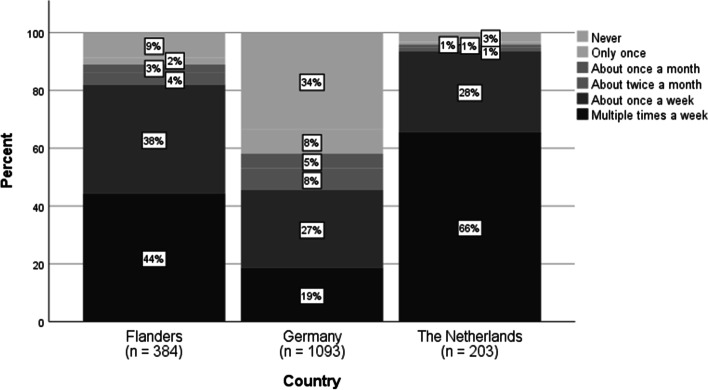
Frequency of contact with students via synchronous formats (item T5_1, *N* = 1680

**Fig. 3:**
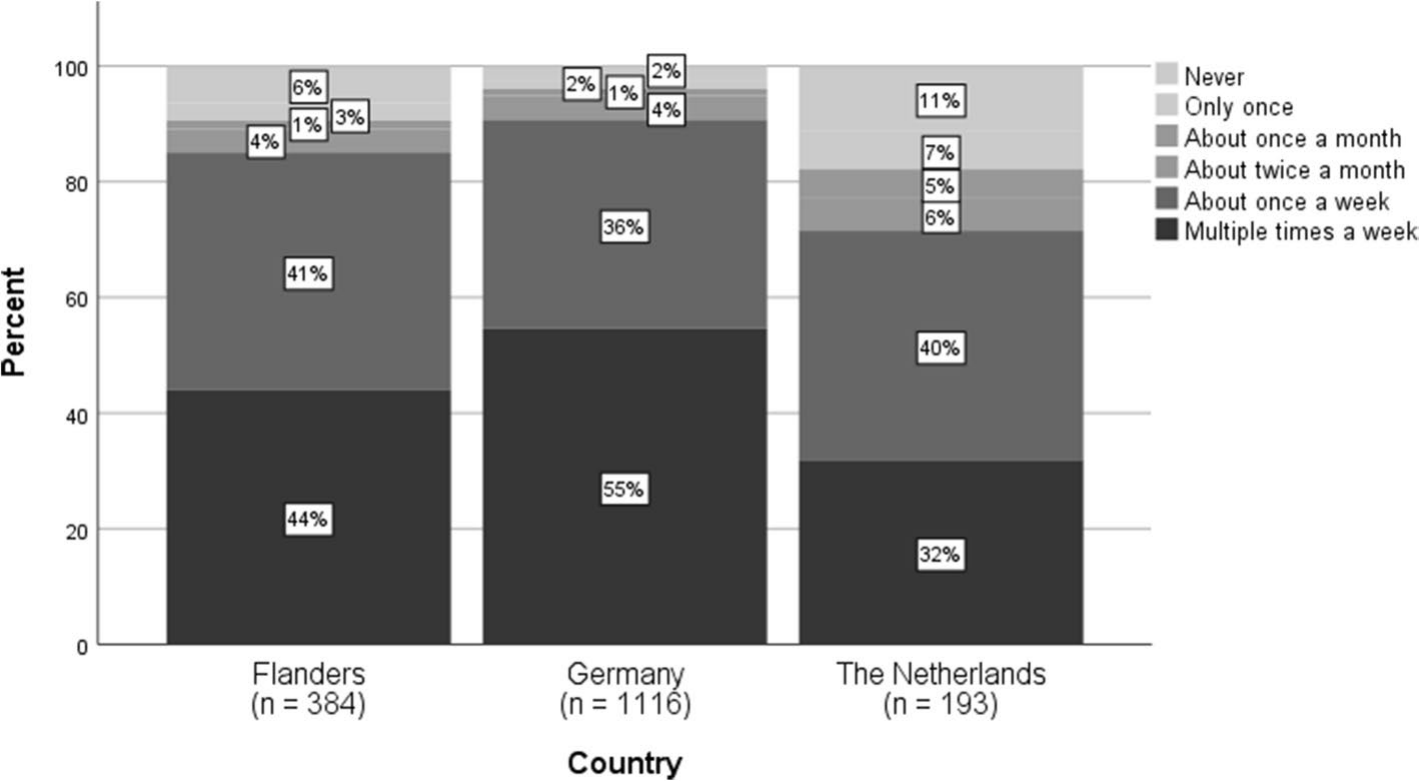
Frequency of contact with students via synchronous formats (item T5_2, *N* = 1693)

When it comes to delivery through asynchronous teaching formats (e.g., forum, sending out exercises via mail), the majority of the teachers (FL: 84%, GE: 92%, NL: 72%) used asynchronous formats weekly after the schools closed. However, the use of synchronous formats (e.g., video conferencing, simultaneous working with students in a shared document, live chats) differed between the countries (see Fig. 2). Remarkably, almost one-third of the German teachers (31%) did not use synchronous formats, compared to 9% in Flanders and 3% in the Netherlands. Only 47% of the German teachers used them every week, compared to 82% of the Flemish and 94% of the Dutch teachers. The use of asynchronous practices per country is shown in Fig. 3.

Most Flemish mathematics teachers (65%) used Smartschool Live, local software available to nearly all schools (item T6) as a video conferencing system to connect with students. In the Netherlands, almost two-thirds (65%) of the mathematics teachers used Microsoft Teams. In Germany, various systems were used such as Microsoft Teams (14%), Zoom (13%), and Jitsi (10%).

For item T7, the majority of the teachers in the Netherlands prepared mathematics lessons at distance by setting up groups for video conferencing (73%), setting up rules for behavior (66%), and giving instructions on how to use the platform (61%). In Flanders, most teachers gave instructions (64%) and set up rules for behavior (53%), and 50% of the Flemish mathematics teachers set up groups for video conferencing. As 40% of the German teachers did not have distant mathematics lessons yet, the overall percentages are lower here: 43% set up rules for behavior, 42% gave instructions on how to use the platform, and 41% set up groups for video conferencing.

**Fig. 4:**
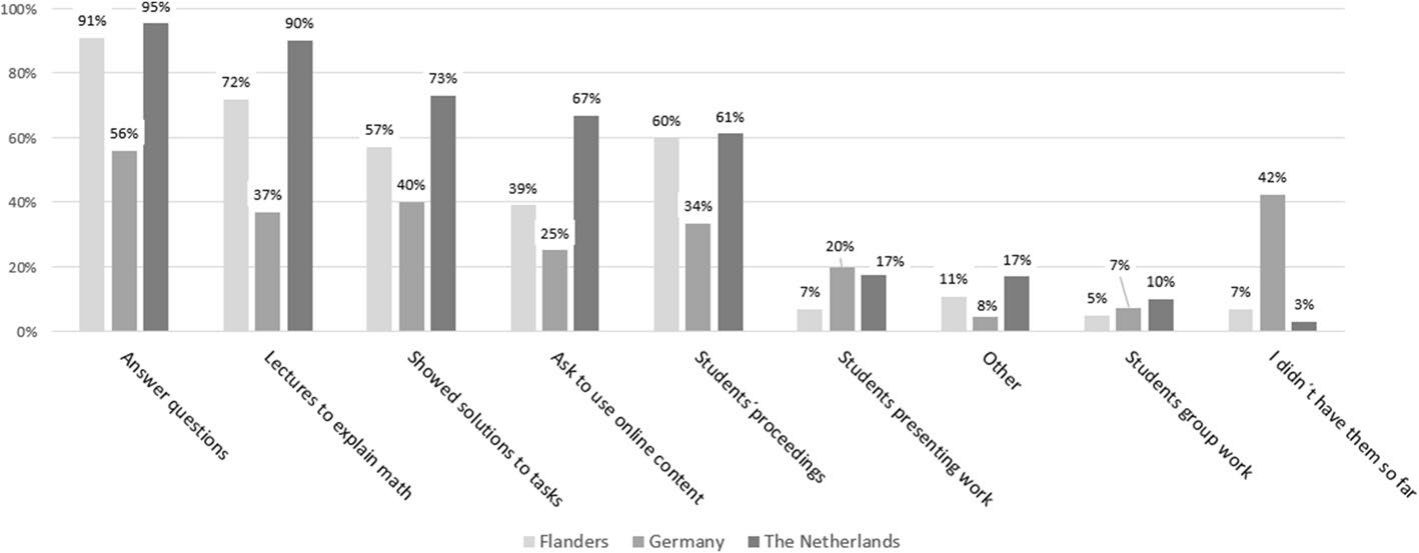
Type of activity during video conferencing lessons (item T8, *N* = 1706)

As a next step, we investigated how teachers used the video conferencing environment during teaching. Figure 4 summarizes the results of item T8. In the three countries, video conferencing lessons are mainly used for answering questions (FL: 91%, GE: 58%, NL: 96%), giving lectures to explain mathematical topics (75%, 39%, 90%, resp.), showing solutions to tasks (59%, 42%, 73%, resp.), asking to use online content (41%, 26%, 66%, resp.), and speaking with students about their progress and their way of working (61%, 35%, 62%, resp.). Activities such as students showing and presenting their work (8%, 21%, 18%) and engaging students in group work (6%, 8%, 10%) are sparse. Once more, the overall percentages for Germany are low here due to the number of German teachers who did not have video conferences so far.

To summarize the findings on teacher practices, the main result is a drastic increase of the use of synchronous formats such as video conferencing (in Germany to a more limited extent), and a decrease in the use of mathematical tools embedded in online exercise platforms and learning environments, as well as of social media.

### Teacher beliefs

As a starting point to consider teachers’ beliefs, item T1 in the questionnaire asks to what extent teachers like working with technology. Item T14 concerns their beliefs on how mathematics education at a distance provides opportunities for specific learning practices. We focused on the following practices: teaching algorithms (T14_1), focusing on mathematical concepts (T14_2), focusing on argumentation and reasoning (T14_3), working with complex mathematical tasks (T14_4), letting students discover mathematics on their own (T14_5), and letting students learn from their own mistakes (T14_6). In addition, one item referred to teachers’ beliefs about digital assessment (T18) and how much these beliefs changed (T19). Furthermore, teachers were asked about their confidence in using digital technology before the closing of schools (T9), their current confidence (T10) and their confidence for using digital formative assessment formats (T20), and the respective change of this confidence (T21). Table 3 gives an overview of the results on these items.

**Table 3:** Mean results on items related to teacher beliefs

Item #	Flanders Mean (SD) *N*	Germany Mean (SD) *N*	The Netherlands Mean (SD) *N*	Total Mean (SD) N
Beliefs				
T14_1: algorithms	4.13 (1.43) 384	4.09 (1.55) 1110	4.14 (1.53) 203	4.11 (1.52) 1697
T14_2: mathematical concepts	3.81 (1.33) 384	3.19 (1.45) 1112	3.66 (1.49) 204	3.38 (1.46) 1700
T14_3: argumentation and reasoning	3.15 (1.45) 384	2.80 (1.43) 1115	3.17 (1.39) 202	2.92 (1.44) 1701
T14_4: complex mathematicaltasks	2.62 (1.31) 384	2.58 (1.41) 1115	2.83 (1.50) 202	2.62 (1.40) 1701
T14_5: discover mathematics on their own	4.22 (1.33) 384	3.72 (1.52) 1122	3.67 (1.49) 203	3.82 (1.49) 1709
T14_6: learn from own mistakes	4.16 (1.31) 384	3.42 (1.48) 1118	3.65 (1.43) 203	3.61 (1.47) 1705
T18: digital assessment can enhance student learning	4.20 (1.34) 384	4.41 (1.33) 1119	3.46 (1.53) 202	4.25 (1.39) 1705
T19: change of T18	3.94 (1.14) 384	3.91 (1.11) 1091	3.11 (1.25) 202	3.82 (1.16) 1677
Confidence				
T9: using digital technology before	54.30 (22.15) 384	58.55 (25.13) 1121	58.33 (22.61) 203	57.57 (24.25) 1708
T10: using digital technology now	75.39 (15.74) 384	71.35 (21.59) 1130	77.48 (15.42) 204	72.98 (19.89) 1718
T20: using digital formative assessment formats	45.56 (28.15) 384	53.70 (27.26) 1099	42.95 (28.01) 192	50.60 (27.87) 1675
T21: confidence change (rescaled to %)	54.84 (23.16) 384	60.24 (23.61) 1087	44.20 (24.11) 200	57.08 (24.13) 1671

T14, 18, 19, 21 on a 1–6 Likert item, e.g., ranging from “Strongly disagree” to “Strongly agree” (see Appendix 1); items T9, 10, 20 on a 1–100 scale

In general, teachers stated that they liked working with technology (T1), and this opinion correlated positively with their confidence (T9): *τ* = 0.42. Teachers’ confidence before and during lockdown increased from 58 (T9) to 73 (T10) on a scale ranging from 0 to 100 and the two correlated (*τ* = 0.60). In particular, of the teachers who were not confident before the schools closed (30% of the sample, scoring less than 50 on T9), 69% indicated being confident during the school closures (T10 ≥ 50). Furthermore, teachers were quite positive on distance learning supporting teaching algorithms (T14_1) and providing means to make students discover mathematics on their own (T14_5). However, teachers were more negative about the opportunities of distance learning for teaching complex mathematical tasks (T14_4). Teachers’ confidence in using digital technology clearly increased during the closing of the schools (T9-T10). Their confidence on using digital formative assessment formats, however, was not very high (T20), and—even more important—increased only to a limited extent (T21).

Country differences appeared: German teachers were less positive about distance learning, particularly for teaching mathematical concepts (T14_2) and argumentation and reasoning (T14_3). In addition, the confidence for using digital technology (T9, T10) of German teachers did not increase as much as in Flanders and the Netherlands. However, German teachers were particularly confident in using digital formative assessment formats (T20). To summarize, the findings suggest positive changes in teachers’ beliefs and confidences.

### Didactical approaches to distance mathematics education

Questionnaire items T13, T16, and T17 concerned the teachers’ didactical approaches. Item T13 asks if mathematics teaching in times of school closure focused on rehearsing and practicing (T13_1), new topics (T13_2), conceptual understanding (T13_3), procedures and algorithms (T13_4), argumentation and reasoning (T13_5), and authentic complex tasks and modeling (T13_6). The results (see Table 4) show that teachers report paying attention to rehearsing, practicing, procedures, and algorithms (T13_1 and T13_4). In the meantime, they also claim a focus on new topics and conceptual understanding (T13_2 and T13_3). Finally, attention to argumentation and reasoning (T13_5) and authentic complex tasks (T13_6) is limited. However, we do not know whether this limited attention is a change compared to regular practices before school lockdown.

**Table 4:** Mean results on the focus in mathematics teaching during lockdown per country (item T13)

Item	Flanders Mean (SD) *n*	Germany Mean (SD) *n*	The Netherlands Mean (SD) *n*	Total Mean (SD) *n*
T13_1: Rehearsing practicing	2.83 (1.55) 384	4.03 (1.56) 1118	1.76 (1.05) 204	3.49 (1.71) 1706
T13_2: New topics	5.29 (0.84) 384	4.22 (1.38) 1128	5.07 (1.01) 204	4.56 (1.33) 1716
T13_3: Conceptual understanding	4.51 (1.21) 384	3.62 (1.42) 1106	4.27 (1.22) 202	3.90 (1.41) 1692
T13_4: Procedures, algorithms	3.95 (1.41) 384	3.97 (1.41) 1104	3.79 (1.44) 202	3.94 (1.41) 1690
T13_5: Argumentation reasoning	2.89 (1.49) 384	2.81 (1.35) 1109	3.30 (1.30) 202	2.89 (1.39) 1695
T13_6: Authentic complex tasks	2.13 (1.32) 384	2.32 (1.32) 1099	2.26 (1.32) 202	2.27 (1.32) 1685

Item on a 1–6 Likert scale, ranging from “Strongly disagree” to “Strongly agree” (see Appendix 1)

**Fig. 5:**
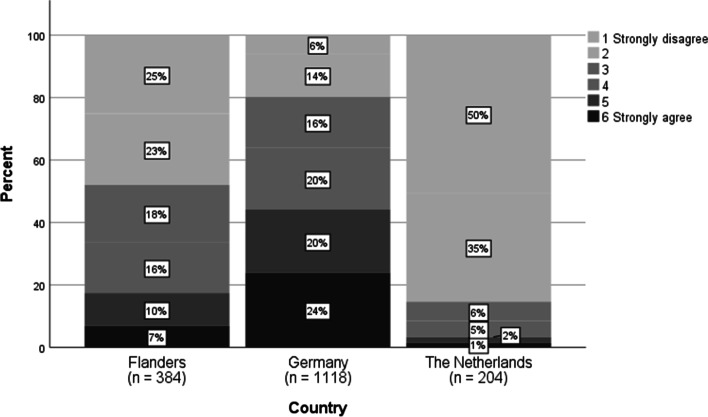
Agreement with the statement “In times of school closure, my math lessons focus on rehearsing and practicing topics that the students already knew” (item T13_1, *N* = 1706)

The results on T13_1 show a remarkable difference between Germany and the other two countries (see Fig. 5). German teachers report more focus on rehearsing and practicing than their colleagues in other countries. An explanation might be the guidelines provided by some German ministries to do so (see Sect. 3.1).

Item T16 investigates interactive formats used in the distant mathematics lessons, and in particular learning intentions and success criteria (T16_1), discussions and tasks (T16_2), feedback (T16_3), peer instruction (T16_4), self-checking (T16_5), and adapting teaching to formative assessment (T16_6). The mean values in Table 5 suggest that the interactive practices did occur on a more or less regular basis, but that giving peers a role in the learning process was only exploited to a limited extent.

**Table 5:** Mean results on interaction formats in mathematics teaching during lockdown (item T16)

Item#	Flanders Mean (SD) *n*	Germany Mean (SD) *n*	The Netherlands Mean (SD) *n*	Total Mean (SD) *n*
T16_1: Learning intentions	3.53 (1.60) 384	3.86 (1.60) 1114	3.64 (1.77) 204	3.76 (1.63) 1702
T16_2: Discussions and tasks	4.22 (1.47) 384	2.94 (1.77) 1111	4.14 (1.57) 203	3.37 (1.78) 1698
T16_3: Feedback	5.07 (0.91) 384	4.52 (1.43) 1117	4.89 (1.20) 202	4.69 (1.32) 1703
T16_4: Peer instruction	3.08 (1.88) 384	2.75 (1.80) 1099	3.02 (1.87) 201	2.86 (1.83) 1684
T16_5: Self-checking	5.23 (1.03) 384	5.02 (1.16) 1117	5.52 (1.00) 202	5.13 (1.13) 1703
T16_6: Formative assessment	3.68 (1.58) 384	3.33 (1.72) 1110	3.06 (1.73) 202	3.38 (1.70) 169

Item on a 1–6 Likert scale, with options Never, Only once, About once a month, About twice a month, About once a week, Multiple times a week (see Appendix 1)

**Fig. 6:**
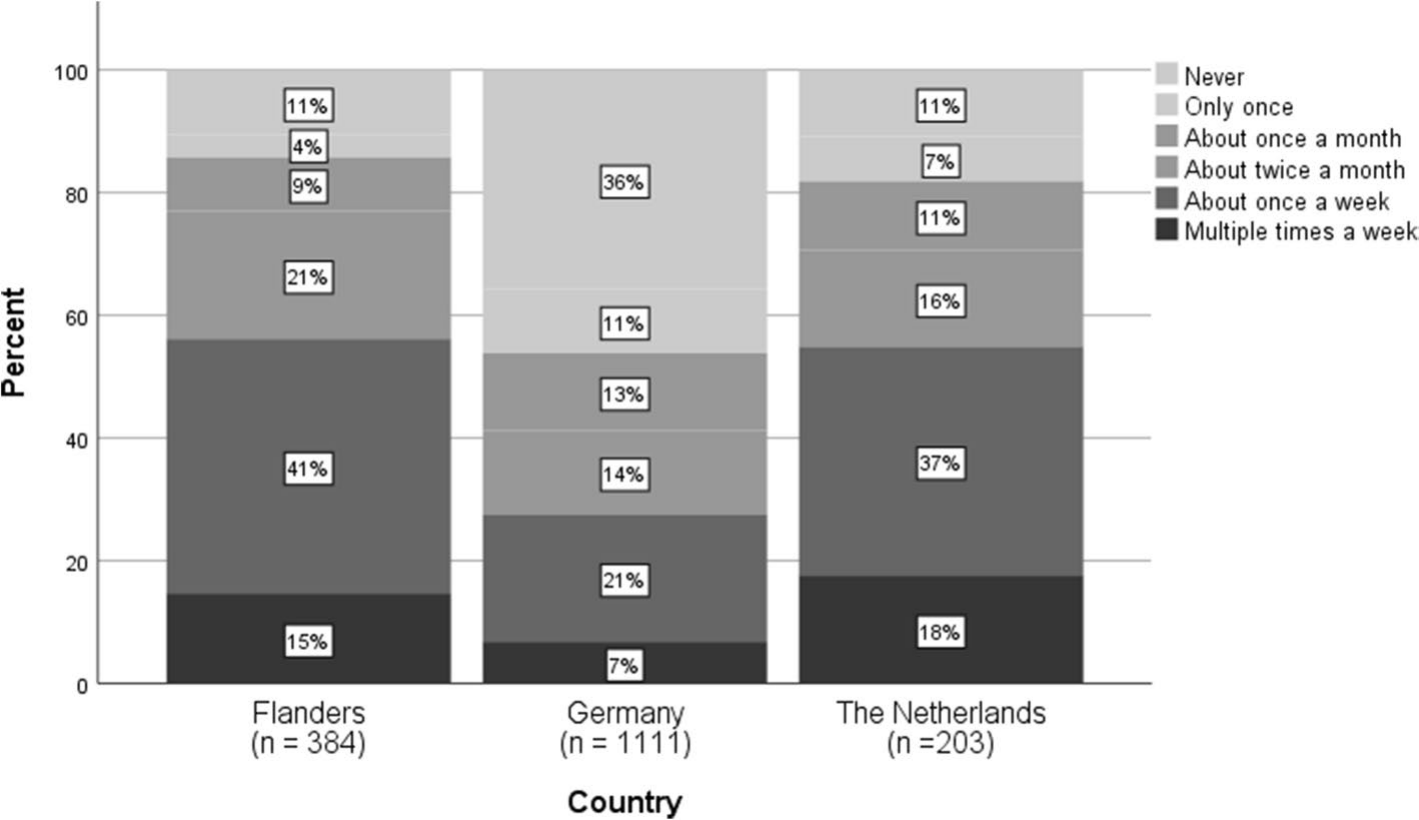
Frequency of discussions and learning tasks that elicit evidence of student understanding (item T16_2, *N* = 1698)

These findings hold for all countries, with the practice of having discussions and tasks to foster conceptual understanding as an exception (T16_2): Fig. 6 shows that German teachers paid little attention to this, probably caused by the limited use of synchronous video conferencing in Germany (see Sect. 4.1).

Quite some attention is spent on activating students as responsible for their learning (Wiliam & Thompson, 2008), especially in the Netherlands (T16_5). However, teachers relatively seldom adapt their own teaching based on the results of formative assessments (T16_6).

To summarize the results on the didactical approach to distance mathematics teaching, the initial hypothesis that teachers would focus on procedures and algorithms and on rehearsing and practicing skills is not confirmed, even if there are some indicators in this direction, in particular for the case of assessment. There is little attention for argumentation and reasoning, and for authentic, complex tasks. Some results suggest that the limited means for interaction through digital technology may hinder didactical approaches, probably mostly in countries where video conferencing is not used frequently, like Germany.

### Assessment

Item T15 asks for the methods the teachers used to keep track of the mathematical learning and as such refers to formative assessment. The following methods are included: gathering written results (T15_1), live questions during video conferences (T15_2), live chat during video conferences (T15_3), assessment questions in the schools’ learning management system (T15_4), online learning environments (T15_5), commercial tutorial systems (T15_6), and audience response systems (T15_7). Table 6 summarizes the results.

**Table 6:** Mean results of methods of formative assessment (item T15)

Item#	Flanders Mean (SD) *n*	Germany Mean (SD) *n*	The Netherlands Mean (SD) *n*		Total Mean (SD) *n*
T15_1: Results through email	5.08 (0.97) 382	4.19 (1.70) 1102	3.27 (1.96) 202	4.28 (1.68) 1687	
T15_2: Live questions in video conference	4.75 (1.56) 382	3.19 (2.02) 1097	4.78 (1.61) 203	3.74 (2.02) 1682	
T15_3: Chat window in video conference	4.04 (2.01) 378	2.49 (1.95) 1072	3.96 (2.09) 200	3.03 (2.11) 1650	
T15_4: Assessment questions in LMS	4.79 (1.37) 382	3.63 (2.14) 1098	3.41 (2.03) 202	3.87 (2.04) 1682	
T15_5: Online learning environment	2.16 (1.63) 379	1.79 (1.44) 1068	1.78 (1.46) 201	1.87 (1.49) 1648	
T15_6: Tasks in tutorial system	1.79 (1.41) 379	1.76 (1.53) 1079	1.96 (1.68) 201	1.79 (1.52) 1659	
T15_7: Task in audience response system	1.53 (1.18) 378	1.34 (0.91) 1077	2.15 (1.68) 198	1.48 (1.12) 1653	

Item on a 1–6 Likert scale with options Never, Only once, About once a month, About twice a month, About once a week, Multiple times a week (see Appendix 1)

The most common method of assessment is gathering results from students (e.g., via email or a cloud system like Dropbox). A less frequently used approach was to keep track of students’ mathematical learning using assessment items in the schools’ learning management system or using live questions during a video conference (either verbally or using the chat window). Even more rare was the use of online learning environments, commercial tutorial systems, or audience response systems. Not surprisingly, formative assessment through video conferencing was used much less by German teachers, compared to their Flemish and Dutch colleagues.

Item T17 investigates didactical aspects of assessment, and specifically addresses procedures and algorithms (T17_1), conceptual understanding (T17_2), argumentation and reasoning (T17_3), and authentic, complex tasks and modeling (T17_4). The results, shown in Table 7, are in line with the findings for item T13 on similar sub-items for teaching: teachers report paying attention to both procedures and algorithms, and to conceptual understanding in their assessment, even if the first seems to gain some more attention than the latter, and in assessment less than in teaching (item T13). This once more nuances our hypothesis that mathematics education at distance might focus on procedural skills at the cost of conceptual understanding. Also, in line with the results of T13 is the limited attention to argumentation and reasoning and to authentic, complex tasks (e.g., modeling tasks) in assessment. All in all, these results are very similar in all three countries.

**Table 7:** Mean results of didactical approaches to assessment (item T17)

Item#	Flanders Mean (SD) *n*	Germany Mean (SD) *n*	The Netherlands Mean (SD) *n*	Total Mean (SD) *n*
T17_1: Executing algorithms and procedures	4.45 (1.41) 371	4.23 (1.60) 1060	4.20 (1.60) 185	4.30 (1.57) 1616
T17_2: Concepts and understanding	4.31 (1.40) 372	3.67 (1.77) 1058	3.78 (1.50) 186	3.83 (1.50) 1616
T17_3: Argumentation and reasoning	2.64 (1.54) 371	2.87 (1.43) 1047	2.88 (1.48) 186	2.82 (1.50) 1604
T17_4: Authentic complex mathematical activity	2.09 (1.42) 371	2.34 (1.80) 1044	2.30 (1.49) 184	2.28 (1.40) 1599

Item on a 1–6 Likert scale ranging from “Not at all” to “Very often” (see Appendix 1)

To summarize, the most-used formative assessment practices in distance mathematics include gathering student materials and video conferencing (with an exception for Germany). Other options, as suggested in the questionnaire, were rarely used. Assessment focused on procedural skills and a little less on conceptual understanding. There is little emphasis on argumentation and reasoning or other demanding activities such as modeling. Taking into account the limited confidence teachers have in their formative assessment skills through digital means (see Sect. 4.2), the overall picture is that formative assessment is an issue in distance mathematics education.

Since we mainly focused on formative assessment, we know little about summative assessment practices. However, summative assessment in the form of high-stake tests was not recommended by the administration in most cases (see Sect. 3.1).

### Crossing boundaries between the four perspectives

In this section, we explore the interplay between the four perspectives that impact teaching practice, that is, instrumental orchestrations, teacher beliefs, didactical approaches, and assessment. To do so, we focus on variables that seem to be the most relevant ones in terms of our hypotheses. For instrumental orchestrations, we include school technical facilities and support (T2) and the use of synchronous and asynchronous teaching formats (T5). For teacher beliefs and confidence, we concentrate on the corresponding items (T1, T9, T10, T14) and include teachers’ previous experience with digital tools (T3). For didactics, we focus on procedural versus conceptual approaches, and practicing old topics versus introducing new topics (T13). Concerning assessment, the items T18 and T20 are included. The overall Kendall’s Tau-*b* correlation matrix for these variables can be found in Appendix 3 (Table 10). In the matrix, values of 0.2 or higher are printed in bold, and the ones of − 0.2 or below in italic.

Let us first consider the interplay between school technical facilities and support on the one hand, and synchronous and asynchronous orchestrations on the other. We would expect the correlation between school facilities and support on the one hand (T2_1 and T2_2) with synchronous distant orchestrations on the other (T5_1) to be important. However, for the three countries together, these correlations are small (Kendall’s Tau-*b* < 0.2). For Flanders and the Netherlands, a possible explanation might lie in the widespread availability of video conferencing software such as Smartschool Live (Flanders) and Microsoft Teams (the Netherlands). For Germany, this correlation is 0.2, which we interpret as modest support for the conjecture that school support did play a role in Germany in facilitating video conferencing.

Second, we wonder how teachers’ beliefs and confidence relate to their (a)synchronous orchestrations and didactical approaches. Teachers’ beliefs are expressed in their appreciation of using digital technology (T1), their prior experience with digital tools (T3), and their prior confidence (T9). Despite our expectations, however, Appendix 3 shows positive but low correlations with synchronous orchestrations (T5_1), and with didactical approaches such as treating new topics under challenging circumstances or focusing on conceptual understanding (T13). As a possible explanation, it might be that teachers, no matter what their previous experience was, were under so much pressure in the lockdown situation that they faced the new challenges, despite their possible doubts or limited experience. Overall, we found little evidence that teachers’ preparation for the new situation in terms of prior views and experiences was decisive in their orchestrations and didactics.

Third, we consider teachers’ beliefs and confidence before school closure and their views on digital assessment. As shown in Table 8, teachers’ general opinion and prior confidence (T1, T9) positively correlated with their views on the opportunities for distance formative assessment (T18) and on their confidence in the ability to use them (T20). We interpret this as a support for the conjecture that teachers’ views on using digital technology in general coincide with their views on distant formative assessment in particular.

**Table 8:** Kendall’s Tau-*b* correlations for teachers’ general opinion (T1) and prior confidence (T9), and opinion on (T18) and confidence in (T20) formative assessment (in bold if > 0.20)

Item#	Country	T9	T18	T20
T1: Enjoying work with digital technology	FL	**0.40**	**0.30**	**0.31**
	GE	**0.43**	**0.27**	**0.35**
	NL	**0.37**	**0.27**	**0.30**
T9: Confidence in using technology before schools closed	FL		0.13	**0.32**
	GE		0.15	**0.40**
	NL		0.17	**0.34**
T18: Digital assessment in mathematics can enhance students’ learning	FL			**0.32**
	GE			**0.27**
	NL			**0.26**
T20: Confidence in using various digital formative assessment formats	FL			
	GE			
	NL			

A tau value above 0.20 is considered moderate, and above 0.30 is considered strong

## Conclusion and discussion

In this study, we investigated which distance teaching practices in secondary mathematics education have emerged in Flanders, Germany, and the Netherlands in the spring 2020 period of school lockdown and how teachers have experienced them. To do so, we designed and administered an online questionnaire among secondary mathematics teachers in the three countries.

In line with the study’s theoretical perspectives, we phrased four sub-questions. With respect to the first one on the distance education practices the mathematics teachers shaped, we found a variety of synchronous and asynchronous practices, with a striking difference on the former: video conferencing was used much more frequently in Flanders and the Netherlands than in Germany. Surprisingly, during this initial phase of school closure, teachers tended to use general digital tools for the delivery of lessons and communication with students more than mathematics-specific learning environments, even though the latter tools had been used before the lockdown. As a possible explanation, we conjecture that the increased attention for new ways to deliver teaching, and synchronous formats in particular, in combination with all organizational changes during the lockdown, may have taken all of teachers’ time and attention at the cost of using specific mathematical tools. The correlations between the use of synchronous orchestrations such as video conferencing and the technological infrastructure and support in schools were limited, in contrast to our expectations. A possible explanation might be that teachers found their own ways to proceed, independent from school infrastructure, for example through using freely available video conferencing software or other freely available resources.

On the second research sub-question on the relation between the emerging practices and the mathematics teachers’ beliefs, we found that teachers’ beliefs played a role in their engagement in distance education, but to a lesser extent than expected. Closely related to teachers’ beliefs seemed to be their confidence in the ability to deal with distance education and the corresponding digital technology. In addition, the distance learning experience increased teachers’ confidence remarkably. In the meantime, the COVID-19 situation made even teachers with limited appreciation for digital technology beforehand engage in distance education, even if there were differences between the three countries.

For the third sub-question on didactical approaches of distance mathematics education, our initial hypothesis was that teachers would focus on “safe and easy” approaches to teaching, that is, on rehearsing and practicing mathematical procedures, rather than on higher-order learning goals such as mathematical understanding and new mathematical topics. The study’s results do not confirm this: teachers report that they paid attention to both rehearsing and practicing and conceptual understanding, and that new topics were also taught. Some differences between countries may be explained by different policies with respect to guiding teachers or not. For example, the ministries’ guidelines in Flanders (initially) and in some states in Germany may have retained teachers from treating new mathematical topics.

Fourth, we addressed assessment formats. We expected teachers to feel somewhat hindered in providing formative feedback to their students through digital means because of the limited means of communication, which would align with the findings by Hodgen et al. (2020). This was confirmed by the teachers’ limited (increase of) confidence in this respect. For formative assessment, teachers made little use of audience response systems, maybe because they were not aware of the possibilities. For summative assessment, the data provide limited information: it seems that many schools may have postponed summative assessment practices, sometimes also on the advice of the ministries of education.

Of course, the four theoretical perspectives act in interplay. We found traces of this interconnection, for example in the correlation between teachers’ general opinions and prior confidences and their confidence in the ability to use opportunities for distance formative assessment.

These conclusions should be interpreted with care because of the study’s limitations. A first limitation is a possible bias in the sample because enthusiastic teachers might have been more willing to reply to the invitation to participate than other colleagues. Also, the sample sizes were quite different for the three countries, even if they reflected the differences in population sizes. A second limitation is the sample of three countries in Western-Europe: one might wonder whether the picture would be different in other parts of the world. A third limitation is that the data are based on teachers’ self-reports. On the topic of paying attention to conceptual understanding during distance lessons, for example, one might wonder whether this is how students perceived it. Fourth and final, the data analysis was done on an item-by-item level rather than using multiple-item-scales. This is in line with the study’s explorative and descriptive character. A confirmative study which includes multiple-item scale analysis would be an important next step.

As a reflection on the study, it is interesting to notice how big the differences between the three countries were, despite them being geographically close and the similar COVID restrictions. Apparently, institutional factors and national educational factors play important roles. Furthermore, we notice that the four perspectives on teaching practices proved valuable and rich. Still, they should not be considered as separate and independent dimensions, but rather as a dynamic system in interplay. Further elaboration of their interconnection would be a challenge for future research.

As a second recommendation for future research, we would be interested in the responses to a similar questionnaire after a longer period, to see whether some findings, such as the limited attention to mathematical tools, would disappear after the novelty of synchronous distance teaching in this “first moment study” is gone, and would be different after some time of familiarization or in a more stable situation.

As a third and final recommendation for future research, students’ perspectives are waiting to be included to get a better view on the real impact of COVID-19 on mathematics education at distance in secondary schools.

## Data Availability

Upon request.

## Appendix 2. Item—research question overview

**Table 9:** Questionnaire item assignements to the research sub-questions

**Item#**	**Research question**			
	**1. Practices**	**2. Beliefs**	**3. Didactics**	**4. Assessment**
T1		X		
T2	X			
T3	X			
T4	X			
T5	X			
T6	X			
T7	X			
T8	X			
T9		X		
T10		X		
T11		X		
T12		X		
T13			X	
T14		X		
T15				X
T16			X	X
T17			X	X
T18		X		
T19		X		
T20		X		
T21		X		

## Appendix 3. Kendall’s Tau-*b* correlation matrix for items possibly relating to teaching practices

**Table 10:** Kendall’s Tau-b correlation coefficients per country, in bold if they are at least 0.20, and italic if they are at highest −0.20

**Item #**		**T1**	**T2_1**	**T2_2**	**T3**	**T5_1**	**T5_2**	**T9**	**T10**	**T13_1**	**T13_2**	**T13_3**	**T13_4**	**T14_1**	**T14_2**
Teaching experience	FL	-.10	.04	.00	-.02	.08	-.02	-.16	-.15	-.13	.11	**.20**	.00	.02	.07
	GE	-.14	-.08	-.10	-.04	-.04	-.05	-.15	-.12	.05	-.12	-.10	.02	-.06	-.14
	NL	-.18	.00	-.05	-.09	.07	-.12	-.18	-.14	.02	-.08	-.06	-.15	.01	-.05
T_1 Enjoying working with technology	FL		**.26**	**.22**	.18	.07	-.03	**.42**	**.46**	.00	.08	.08	.10	**.30**	**.25**
	GE		**.22**	**.21**	.**29**	.16	.08	**.43**	**.44**	-.04	.11	.11	.06	.14	.18
	NL		**.32**	**.26**	.**35**	.18	.02	**.39**	**.47**	.01	.02	.06	.07	**.22**	**.33**
T2_1 Technical facilities	FL			**.68**	.16	.02	-.05	**.21**	**.20**	-.03	.12	.09	.04	.14	.19
	GE			**.66**	.**23**	.18	.10	**.21**	**.27**	-.08	.14	.08	.05	.12	.12
	NL			**.67**	.18	.02	-.03	.14	**.31**	-.19	.12	.07	-.02	**.21**	.17
T2_2 Technical support	FL				.09	-.01	-.07	.19	.17	-.03	.10	.02	.05	.09	.09
	GE				**.21**	**.20**	.10	**.20**	**.27**	-.06	.16	.11	.05	.10	.13
	NL				.10	.03	.05	.10	**.24**	*-.20*	.16	.13	.02	**.23**	.17
T3 Total	FL					.11	.06	**.30**	**.22**	.03	-.05	.04	.02	.12	.12
	GE					.**21**	.11	**.35**	**.34**	-.02	.02	.09	.05	.11	.13
	NL					.09	.07	**.32**	**.27**	.08	-.13	-.04	.03	.13	.14
T5_1 Use synchronous formats	FL						-.02	.06	.10	-.03	.05	.13	.00	.14	.13
	GE						.11	.11	**.23**	-.09	.15	.14	.05	.10	.16
	NL						-.05	.11	.17	.08	-.02	.07	.03	**.20**	.14
T5_2 Use asynchronous formats	FL							.00	.03	.02	.06	.02	-.02	.01	.04
	GE							.08	.13	-.05	.13	.09	.10	.13	.08
	NL							.03	.06	-.06	-.03	.00	-.02	.03	.02
T9 Confidence digital technology before schools closed	FL								**.58**	.01	.02	.06	.05	.11	.09
	GE								**.63**	-.01	.07	.10	.05	.10	.14
	NL								**.55**	-.01	.00	.03	.01	.05	.09
T10 Confidence digital technology since schools closed	FL									.03	.09	.09	.06	.15	.13
	GE									-.06	.14	.14	.08	.16	**.20**
	NL									-.10	.07	.06	.06	.12	.16
T13_1 Topics already known	FL										-.19	-.03	.10	-.06	.01
	GE										*-.33*	-.15	-.03	-.06	-.06
	NL										*-.44*	-.14	.08	-.03	-.07
T13_2 New topics	FL											**.40**	.19	**.21**	**.22**
	GE											**.38**	.17	.18	**.23**
	NL											**.30**	**.20**	.15	.12
T13_3 Conceptual / deep understanding	FL												**.22**	**.20**	**.37**
	GE												**.21**	.16	**.45**
	NL												**.29**	.19	**.36**
T13_4 Procedures / algorithms	FL													**.22**	.15
	GE													**.42**	.15
	NL													.17	.09
T14_1 Beliefs procedures	FL														**.45**
	GE														**.38**
	NL														**.56**
T14_2 Beliefs concepts	FL														
	GE														
	NL														

## Footnotes

1 www.qualtrics.com
